# Predictors of health care practitioners’ normative attitudes and practices towards sexual and reproductive health and rights: a cross-sectional study of participants from low-income countries enrolled in a capacity-building program

**DOI:** 10.1080/16549716.2020.1829827

**Published:** 2020-10-20

**Authors:** Gilbert Tumwine, Anette Agardh, Christina Gummesson, Pius Okong, Per-Olof Östergren

**Affiliations:** a Social Medicine and Global Health, Department of Clinical Sciences, Lund University, Malmö, Sweden; b Department of Obstetrics and Gynecology, St. Francis Hospital Nsambya, Kampala city, Uganda; c Centre for Teaching and Learning, Faculty of Medicine, Lund University, Lund, Sweden

**Keywords:** Health care practitioners, SRHR attitudes, SRHR behaviours, normative attitudes, normative behaviours, low-income countries

## Abstract

**Background**: Sexual and Reproductive Health and Rights (SRHR) is a concept of human rights applied to sexuality and reproduction. Suboptimal access to SRHR services in many low-income countries results in poor health outcomes. Sustainable development goals (3.7 and 5.6) give a new impetus to the aspiration of universal access to high-quality SRHR services. Indispensable stakeholders in this process are healthcare practitioners who, through their actions or inactions, determine a population’s health choices. Often times, healthcare practitioners’ SRHR decisions are rooted in religious and cultural influences. We seek to understand whether religious and cultural influences differ significantly according to individuals’ characteristics and work environment.

**Objective**: The purpose of this study was to examine the role of healthcare practitioners’ individual characteristics and their work environment in predicting normative SRHR attitudes and behaviours (practices). We hypothesized that religion and culture could be significant predictors of SRHR attitudes and practices.

**Methods**: A quantitative cross-sectional study of 115 participants from ten low-income countries attending a capacity-building programme at Lund University Sweden was conducted. Linear regression models were used to assess for the predictive values of different individual characteristics and workplace environment factors for normative SRHR attitudes and SRHR practices.

**Results**: Self-rated SRHR knowledge was the strongest predictor for both normative SRHR attitudes and normative SRHR practices. However, when adjusted for other individual characteristics, self-rated knowledge lost its significant association with SRHR practices, instead normative SRHR attitudes and active knowledge-seeking behaviour independently predicted normative SRHR practices. Contrary to our hypothesis, importance of religion or culture in an individual’s life was not correlated with the measured SRHR attitudes and practices.

**Conclusion**: Healthcare practitioners’ cultural and religious beliefs, which are often depicted as barriers for implementing full coverage of SRHR services, seem to be modified by active knowledge-seeking behaviour and accumulated working experience with SRHR over time.

## Background

Sexual and Reproductive Health and Rights (SRHR) is a concept of human rights applied to sexuality and reproduction.

Access to high-quality SRHR services in many low-income countries remains suboptimal and has led to persistently poor health outcomes [1–3]. Sustainable Development Goals (SDGs) 3.7, ‘By 2030, ensure universal access to sexual and reproductive health-care services, including for family planning, information and education, and the integration of reproductive health into national strategies and programmes’, and 5.6, ‘Ensure universal access to sexual and reproductive health and reproductive rights’, give a new impetus to the necessity of achieving universal access to SRHR by 2030 [1].

Healthcare practitioners are indispensable stakeholders in healthcare delivery. As healthcare planners, they determine which services are offered in a country, and as program officers or service providers, they influence how these services are implemented. Hence, they influence a population’s health choices and the quality of the services provided [1,4,5]

Health practitioners’ behaviours and attitudes, often linked to religious and/or cultural values [6–9] or self-efficacy (in knowledge and skills) influence the content and the quality of care [10,11]. For example, despite global consensus that contraception is a relatively cheap and effective option to prevent unplanned pregnancies [1,12] and reduce maternal death, some health practitioners still find it unacceptable to provide [9]. Religious and/or cultural values have been reported as major influences on teachers’ and healthcare practitioners’ unwillingness to discuss SRHR matters with young people [13–15] or refusal to provide abortion services [6]. Attitudes towards provision of SRHR services have been linked to healthcare workers’ levels of education and appropriate SRHR training in many low-income countries. For example, nurses that were more educated in adolescent sexuality held positive attitudes towards sexual and reproductive health needs of young people in Kenya and Zambia [16], health workers’ positive attitudes towards emergency contraception were positively linked to having had family planning education update in the preceding year in Uganda [17] and, in Kenya, teachers in public schools who lacked training were less comfortable in addressing AIDS education in public schools [13].

A number of theories have been used to explain how knowledge translates into practice or impacts on attitudes and behaviours. Some of these theories include the Knowledge, Attitudes and Practice (KAP) theory [18] and the Transtheoretical Model [19]. The KAP theory postulates that attitudes influence behaviour (practices), but can be modified by new knowledge. The Transtheoretical Model (TTM) describes the process of intentional behavioural change. It suggests that people transition through defined stages when altering from undesirable to desirable behaviours, depending on an interplay between individual’s characteristics and their environment. Healthcare practitioners’ actions towards clients’ needs are a form of intentional behaviour with a spectrum of attributes ranging from total inaction to pro-active provision of health services that often are not allowed, by law or societal norms, in their settings. The influences of religion, culture, and self-efficacy (in knowledge) on healthcare practitioners’ SRHR attitudes and behaviours have been well established. However, what is not very well known is whether these influences differ significantly according to individual characteristics such as age, gender, work experience with SRHR, and work environment.

### Objective

The purpose of this study was to examine the role of healthcare practitioners’ individual characteristics and their environment in predicting normative SRHR attitudes and behaviours (practices). Specifically, the study examined age, gender, education, self-rated SRHR knowledge and the importance attached to religion and culture as individual characteristics, whereas the influence of religion and culture, work experience in years, and sector and level of employment were considered as the health practitioners’ environment. The goal was to determine which characteristics are predictive of normative SRHR attitudes and behaviours and potentially amenable to targeted interventions for behavioural change among health care practitioners in low- and middle-income countries. We hypothesized that religion and culture could be significant predictors of normative SRHR attitudes and practices.

## Methods

### Setting

This study was conducted at the beginning of the four-week international training program (ITP) taking place at Lund University, Sweden. The program was commissioned by the Swedish International Development Cooperation and aimed at improving access to quality SRHR services in low-income countries through a rights-based framework [20]. A central component of the ITP program was to improve knowledge about current international SRHR policies in order to facilitate positive attitudes and behaviours towards different aspects of SRHR among the participants.

### Sample

Participants in this study were healthcare practitioners working in medical facilities and civil society organizations in private and public sectors in low-income countries. They were purposively selected to participate in the ITP, because their individual profiles and the positions they held suggested that they were influential decision makers in their respective health systems. Participants were from Ethiopia, Zambia, South Sudan, Zimbabwe, Bangladesh, Uganda, Kenya, Tanzania, Myanmar and Liberia. Each country team consisted of 4–6 members. They included male and female individuals, midwives, nurses and doctors, middle-level managers, and policy makers. A total of 115 health practitioners participating in the ITP were enrolled in this study, 58 in October 2017 and 57 in October 2018.

### Study design

A quantitative cross-sectional study design was used. To determine participants’ individual characteristics, attitudes and practices, a self-administered structured questionnaire was used. The questionnaire contained items regarding the participants’ age, gender, level of education, sector and category of employment, and number of years working with SRHR at the time of the study. In addition, the questionnaire was used to assess self-rated SRHR knowledge, normative SRHR attitudes and normative SRHR practices. The elements assessing knowledge, attitudes and practices were developed based on a combination of the Knowledge Attitudes and Practices (KAP) model and the Transtheoretical Model (TTM). The questionnaire was piloted in a non-study sample consisting of two master’s students in the Public Health programme at Lund University (SRHR healthcare practitioners from low-income countries) and necessary adjustments were made before the study was conducted.

### Procedure

The first and the second authors approached the participants in a group to provide information about the study, explain the study objectives and ask for their participation. Written informed consent was obtained according to the principles of Helsinki declaration [21]. A detailed consent form is available as supplementary information. A self-administered questionnaire was filled in on the first day of the training programme-precisely after the introductions and before any program content was introduced to the participants. The study was conducted in English, which is the language of instruction in the training program. A total of 115 participants completed the questionnaire, representing 100% of the ITP participants. The study was approved by the Regional Ethical Review Board in Lund, Sweden, and given ethical approval number DNR 2017/823. No compensation was given for participating in this study.

### Independent variables

The independent variables were participants’ individual characteristics and SRHR work environment.

Individual characteristics were age, gender, education, self-rated knowledge and perceived importance of religion or culture in one’s life. Age was reported as ‘equal or less than 40 years’ or ‘more than 41’ (reference category) and gender as ‘male’, (reference category), ‘female’ or ‘other’. Level of education was defined as ‘completed high school or its equivalent’, ‘completed bachelor’s degree or its equivalent’ (reference category), ‘completed master’s degree or its equivalent’, or ‘completed doctorate/PhD or its equivalent’. SRHR self-rated knowledge was assessed on the basis of the participants’ responses to the following question, “How do you rate your knowledge regarding the following items (components of SRHR included in ITP): ‘comprehensive sex education’, ‘contraception’, ‘abortion’, ‘cervical cancer screening’, ‘the lesbian, gay, bisexual and trans-gender community’s health’, ‘sexual orientation and gender identity’, ‘sexual coercion and violence’, and ‘health policy regarding SRHR?’ The responses for each of the items were coded on a scale of 1–5, where 1 = Very Low, 2 = Low, 3 = Neither high nor low, 4 = High and 5 = Very High. Each participant’s responses to each of the SRHR aspects were summed up to yield a composite score for the SRHR self-rated knowledge variable. Scores equal to or less than the mean were categorized as ‘low self-rated knowledge’ (reference category) and scores greater than the mean as ‘high self-rated knowledge.’ This scale was developed for the purposes of this study. In addition, participants were asked to respond with ‘yes’ or ‘no’ to the questions, ‘Does religion play an important role in your life?’ and ‘Does culture/tradition play an important role in your life?’

SRHR work environment was assessed as sector of employment, level of employment, area of operation, working years with SRHR and perceived influence of religion and culture on SRHR decision making. Sector of employment was defined as either ‘public health sector’, ‘private health sector’, ‘public education sector’, ‘private education sector’, ‘non-governmental organization’ or ‘other’. The sector of employment was aggregated into two categories, ‘public sector’ consisting of public health and public education sectors and ‘private sector’ (reference category) consisting of private education and private health sectors, non-governmental organizations and ‘others’. Level of employment was defined as ‘senior management’, ‘program officer’, ‘service provider’, or ‘others’. Two aggregated categories were created for this variable; ‘senior management and program officers’ and ‘service providers, and others’ (reference category). Area of operation was reported as ‘local’, ‘intermediate/regional’, and ‘national’ (reference category). Experience of working with SRHR was reported as number of years, and dichotomized as ‘seven years or less’ and ‘eight years or more’, based on mean of seven years. The influence of religion and the influence of culture on SRHR decisions making were assessed by the following questions, to which participants responded with either ‘yes’ or ‘no’: ‘Does religion influence your decision making with regard to SRHR?’, ‘Does culture/tradition influence your decision making with regard to SRHR?’.

### Dependent variables

The outcome variables were normative SRHR attitudes, active SRHR knowledge-seeking and normative SRHR practice.

To assess normative SRHR attitudes, participants were asked to indicate their level of agreement with the following statements:

‘I believe that abortion is a woman’s right’, ‘I believe that young people should have access to contraception’, ‘I believe that all young people should have access to comprehensive sex education’, ‘I believe that the LGBT community should have equal access to HIV/STI care like anyone else’, ‘I believe that both men and women are affected by sexual violence’, ‘I believe that sexual orientation and gender identity is a human right’, and ‘I believe that inequality is responsible for poor maternal and neonatal health outcomes’. The responses were coded on a scale of 1–5, where 1 = Strongly disagree, 2 = Disagree, 3 = Not sure, 4 = Agree and 5 = Strongly agree. Each participant’s responses from the above questions were summed up to yield a composite score for the variable ‘normative SRHR attitudes’. High SRHR attitude scores were interpreted as having ‘normative attitudes’ towards SRHR as defined in international policy guidelines [1], while low-attitude scores were interpreted as having ‘non-normative’ attitudes. This scale was developed for the purposes of this study.

The normative SRHR practice and active SRHR knowledge-seeking practice instruments were developed based on constructs derived from the Transtheoretical Model (TTM) [19]. The Transtheoretical Model describes change in behaviour as a deliberate process that happens over time through a cyclic process of 5 stages; pre-contemplation, contemplation, preparation, action, and maintenance stages. In the pre-contemplation stage, individuals have no intention to act within a foreseeable time, are often not mindful of the negative effect of their behaviour and undervalue the advantages of changing. In the contemplation stage, individuals have an intention to take action and acknowledge that their behaviour may have negative consequences but are still hesitant about change. Individuals in the preparation stage are ready to act often in small steps, while, in the action stage, individuals have recently taken action and intend to keep moving forward. During maintenance stage, individuals have continued their behavioural change for a while and intend to uphold the behaviour.

The Transtheoretical Model is constructed on the belief that behavioural change is a process that involves a common set of change processes that are almost similar across a broad range of health behaviours and has been used in interventional studies to encourage adaptation of healthy behaviours such as smoking cessation, engaging in active lifestyles, and choosing of healthy nutritional diet. Its utility in understanding decision-making process has been reported [22,23].

Active SRHR knowledge-seeking was defined as an individual’s intention to seek more knowledge about enhancing access to SRHR services. This was assessed by the following question: “What are your thoughts about getting more knowledge concerning the following: ‘abortion’, ‘cervical cancer screening’ ‘youth access to contraception’, ‘youth access to comprehensive sex education’, ‘health policy regarding SRHR’, ‘LGBT community’s health needs’, ‘sexual coercion and sexual violence’ and ‘sexual orientation and gender identity’?

The responses for each component were ordered on a scale of 1–5 as follows:

1 = ‘I have not thought about seeking out more information in the next 6 months’,

2 = ‘I am considering seeking out more information in the next 6 months’,

3 = ‘I have decided to take steps to gain more information in the next 30 days’,

4 = ‘I have taken steps to acquire more knowledge in the last 6 months, not including ITP’,

5 = ‘I have taken steps to acquire more knowledge for more than 6 months, not including ITP’.

Each participant’s responses to the questions corresponding to the different SRHR components were summed up to yield a composite score for the variable active SRHR knowledge-seeking practice. High scores were interpreted as ‘active knowledge seeking’ and low scores as ‘less active knowledge-seeking’ practice that enhances access to SRHR services.

Normative SRHR practice was defined as individuals’ intention to enhance access to SRHR services. It was assessed from participants’ responses to the following question, “Which of these statements best describes your behaviour towards the following: ‘access to abortion’, ‘cervical cancer screening’, ‘youth access to contraception’, ‘youth access to comprehensive sex education’, ‘equal access to health’,‘LGBT community and their access to HIV/STI care’ and ‘sexual violence’? Participants were asked only to respond to questions corresponding to the aspects of SRHR that they had ever worked with. The responses to each aspect were ordered on a scale of 1–5 corresponding to the stages of change according to the Transtheoretical Model: 1 = Pre-contemplation (‘Is not something I have thought about’), 2 = Contemplation (‘Is something I have thought about as being important’), 3 = Preparation (‘I have decided to take steps about it’), 4 = Action (‘I have taken steps in the last 6 months about it’), 5 = Maintenance (‘I have taken steps for more than 6 months about it’). Each participant’s responses were summed up to yield a composite score for the variable normative SRHR practice. High scores were interpreted as ‘more likely to take steps’ i.e. normative SRHR practices towards improving access to sexual and reproductive health and rights and low scores as ‘less likely to take steps’ or non-normative.

## Statistical analysis

Data were analysed using SPSS version 24. Scores of SRHR self-rated knowledge, normative SRHR attitudes, active SRHR knowledge-seeking, and normative SRHR practice were assessed for normality and the mean, median and standard deviations determined. Bivariate linear regression was used to examine the association between individual characteristics and work environment, and participants’ scores regarding normative SRHR attitudes, active SRHR knowledge-seeking, and normative SRHR practices, respectively.

Predictor variables were tested for multicollinearity. Multivariate linear regression was used to examine the association between: (i) influence of religion and the three outcome variables, i.e. normative SRHR attitudes, active SRHR knowledge seeking, and normative SRHR practice, and (ii) influence of culture and the three outcomes variables. Three models were used to adjust for individual characteristics and other work environment variables. Model 1 adjusted for age and gender, model 2 adjusted for age, gender, education, years working with SRHR and employment sector, and model 3 adjusted for age, gender, education, years working with SRHR, employment sector and self-rated knowledge. The association between normative SRHR attitudes and normative SRHR practices was examined by linear regression, adjusting for age, gender, active SRHR knowledge-seeking, education and SRHR self-rated knowledge. Statistical interaction analyses were conducted to determine whether the influence of religion or culture on active knowledge seeking and normative practice varied with the individual characteristics and work environment. Using linear regression, main effects and interaction effects were determined. Statistical significance was accepted at p < 0.05. Missing data were excluded (pairwise) from cases required for any analysis.

To determine whether assumptions of linear regression were violated, residual diagnostics were conducted for normality of residuals using a histogram and QQ plots. Cronbach alpha test [24] was done to determine the internal consistency of the items used to measure the outcome variables and the internal consistency of the scale was considered to be good if the score was ≥ 0.7.

## Results

The study had 115 participants, 46 males and 69 females, of which two thirds were service providers and one third worked as program managers or officers. About half of the participants had been working with SRHR for eight years or more, and the majority of them had attained a Bachelor’s degree or more. The minimum score from the 8 items assessing self-rated knowledge was 8 and maximum was 40 and participants’ scores ranged from 13 (lowest score) to 40 (highest score). The mean score was 31 (SD = 5) and 44% had scores higher than the mean. Most of the participants, 58%, were employed in public sectors. Although 89% indicated that religion played an important role in their lives, only 45% reported that religion influenced their decision making as SRHR practitioners. Likewise, 69% indicated that culture/tradition played an important role in their lives but only 42% thought that culture/tradition influenced their SRHR decisions (Table 1).

**Table 1.** Participants’ characteristics

		Number (Total N = 115)	Percent (%)
Gender	Male Female Missing	46 69-	40.0 60.0-
Age	40 years or less 41 years or more Missing	67 48-	58.3 41.7-
Years working with SRHR	Seven years or less Eight years or more Missing	62 45-	53.9 39.1-
Education	Completed high school Bachelor’s degree Master’s degree Doctorate Missing	8 56 43 8-	7.0 48.6 37.4 7.0-
Area of operation	Local and intermediate National Missing	37 78-	32.2 67.8-
Employment level	Program officers and managers Service providers Missing	40 74 1	34.8 64.3 0.9
Employment sector (health and education)	Public sector (hospitals, schools and agencies) Private sector (hospitals, schools and NGOs) Missing	67 48-	58.3 41.7-
Influence of religion	Yes No Missing	52 63-	45.2 54.8-
Importance of religion	Yes No Missing	102 13-	88.7 11.3-
Influence of culture	Yes No Missing	48 67-	41.7 58.3-
Importance of culture	Yes No Missing	79 35 1	68.7 30.4 0.9
SRHR self-rated knowledge	Low High Missing	61 50 4	53.0 43.5 3.5

The Cronbach alpha scores were very high (0.88 and 0.89, respectively) for active SRHR knowledge-seeking and normative SRHR practice, indicating very good internal consistency, and the alpha score was good (0.66) for the normative SRHR attitude scale. The item by item analysis showed that the internal consistency did not improve if any items were excluded from any of the scales, and therefore all the original items were kept in the scale construction in the analyses (Table 2). All the three outcome variable scores were normally distributed and hence linear regression was used as the main method of analysis.

**Table 2.** Normative SRHR practice, normative SRHR attitudes and active SRHR knowledge-seeking scales’ reliability test

Domain	Corrected item total correlation	Cronbach Alpha if item is deleted	Cronbach alpha
**Normative SRHR practice (7 items)**			0.89
Which of these statements best describes your behaviour towards access to abortion	0.47	0.91	
Which of these statements best describes your behaviour towards access to cervical cancer screening	0.62	0.89	
Which of these statements best describes your behaviour towards youth access to contraception	0.83	0.87	
Which of these statements best describes your behaviour towards youth access to comprehensive sexuality education	0.71	0.88	
Which of these statements best describes your behaviour towards equal access to health	0.85	0.86	
Which of these statements best describes your behaviour towards LGBT community and their access to HIV/STI care	0.61	0.89	
Which of these statements best describes your behaviour towards sexual violence	0.79	0.87	
**Normative SRHR attitudes (7 items)**			0.66
I believe that abortion is a woman’s right	0.47	0.61	
I believe that all young people should have access to contraception	0.43	0.61	
I believe that all young people should have access to comprehensive sexuality education	0.42	0.63	
I believe that the LGBT community should have access to HIV/STI care like anyone else	0.37	0.64	
I believe that both men and women are affected by sexual violence	0.33	0.64	
I believe that sexual orientation and gender identity is a human right	0.53	0.58	
I believe that inequality is responsible for poor maternal and neonatal health outcomes	0.17	0.68	
**Active SRHR knowledge seeking (8 items)**			0.88
What are your thoughts about getting more knowledge concerning access to abortion	0.70	0.86	
What are your thoughts about getting more knowledge concerning cervical cancer screening	0.67	0.87	
What are your thoughts about getting more knowledge concerning youth access to contraception	0.62	0.87	
What are your thoughts about getting more knowledge concerning youth access to comprehensive sexuality education	0.56	0.88	
What are your thoughts about getting more knowledge concerning health policy regarding SRHR	0.70	0.86	
What are your thoughts about getting more knowledge concerning LGBT community’s health needs	0.55	0.88	
What are your thoughts about getting more knowledge concerning sexual coercion and sexual violence	0.72	0.86	
What are your thoughts about getting more knowledge concerning Sexual orientation and gender identity	0.69	0.87	

The minimum score from the 7 items assessing SRHR attitudes was 7 and the maximum was 35. Participants’ scores ranged from 21 to 35 with a mean of 31 (SD = 3). The minimum score from the 8 items assessing active SRHR knowledge-seeking practice was 8 and maximum was 40 and participants scores ranged from 11 to 40 with a mean score of 25 (SD = 7) and the minimum possible score from the 7 items assessing normative behaviour was 7 and 35 was the maximum and participants scores ranged from 8 to 35 with a mean of 16 (SD = 8). Bivariate linear regression analysis was performed to determine the association between participants’ characteristics, the work environment and the three outcome variables, Table 3. Reporting that religion did not influence one’s SRHR decisions and high self-rated SRHR knowledge were the only independent variables that were significantly associated with high (normative) SRHR attitude scores. On the other hand, more years working with SRHR, being a program officer/manager, employment with the public (in health or education), being uninfluenced by religion, being uninfluenced by culture, and high self-rated knowledge were significantly associated with a high score concerning active SRHR knowledge-seeking. A high number of years working with SRHR, employment within the public sector (in health or education), and high self-rated knowledge of SRHR were all significantly associated with high normative SRHR practice scores. However, there was no statistically significant association between participants’ age, gender or level of education with any of the outcome variables. Also, there was no significant association between religious or cultural influence and SRHR decisions regarding SHHR normative practice scores in the bivariate linear regression analysis.

**Table 3.** Association, by means of bivariate linear regression, between participants’ characteristics and normative SRHR attitudes, active SRHR knowledge seeking and normative SRHR practice scores

	Normative SRHR attitudes scores B (95% CI)	Active SRHR knowledge-seeking scores B (95% CI)	Normative SRHR practice scores B (95% CI)
Gender, Male (Ref: Female)	−0.14 (−1.28–1.00)	−0.64 (−3.21–1.93)	0.21 (−2.83–3.25)
Age 40 years or less (Ref: 41 years or more)	0.39 (−0.74–1.53)	0.77 (−1.78–3.32)	0.41 (−2.61–3.43)
More years working with SRHR (Ref: Less than 7)	0.05 (−0.04–0.15)	0.25* (0.03–0.46)	0.36** (0.11–0.61)
Higher education (Ref: Bachelor’s degree)	0.33 (−0.45–1.11)	1.18 (−0.59–2.95)	−0.56 (−2.6–1.49)
Local level employment (Ref: National level)	0.49 (−0.21–1.19)	1.42 (−0.17–3.00)	0.07 (−1.99–1.85)
Employment as officers & managers (Ref: Service providers)	0.31 (−0.25–0.88)	1.45* (0.22–2.67)	1.15 (−0.34–2.65)
Public sector employment (Ref: Private sector employment)	0.29 (−0.00–0.59)	0.82* (0.18–1.46)	0.84* (0.08–1.60)
Religion not important in life (Ref: Important)	0.90 (−0.93–2.74)	1.29 (−2.62–5.20)	−0.85 (−5.54–3.85)
No influence of religion (Ref: Influence)	1.95*** (0.88–3.02)	3.11* (0.63–5.58)	1.61 (−1.36–4.59)
Culture not important in life (Ref: Important)	1.06 (−0.14–2.26)	0.32 (−2.4–3.01)	0.41 (−2.83–3.64)
No influence of culture (Ref: Influence)	0.97 (−0.16–2.10)	2.69* (0.15–5.22)	2.77 (−0.20–5.74)
High SRHR self-rated knowledge (Ref: Low self-rated knowledge)	0.17** (0.07–0.27)	0.66*** (0.46–0.86)	0.53*** (0.26–0.80)

B: Unstandardized coefficient, P value = *<0.05, **<0.001, ***<0.000,

Table 4 (a) shows the association between influence of religion on SRHR decision-making and the three outcome variables when adjusted for age, gender, education, years working with SRHR, employment sector, and self-rated knowledge at multivariate analysis. The absence of religious influence and high self-rated knowledge remained significantly associated with normative SRHR attitude scores. However, although the absence of religious influence was positively associated with high active SRHR knowledge-seeking scores in the unadjusted analysis, (Table 3), the association was no longer significant in the fully adjusted model 3 (Table 4a). Years working with SRHR and high self-rated SRHR knowledge remained positive predictors of active SRHR knowledge-seeking scores, in addition to level of education. More years working with SRHR and high self-rated knowledge were the only variables significantly associated with normative SRHR practice scores in the fully adjusted model at multivariate analysis (Table 4a).

**Table 4.** (a): Association by means of linear regression, between influence of religion on SRHR decisions and normative SRHR attitudes, active SRHR knowledge seeking and normative SRHR practice scores adjusted for participants’ characteristics

	Normative SRHR attitudes score				Active SRHR knowledge-seeking score			Normative SRHR practice score		
	Model 1		Model 2	Model 3	Model 1	Model 2	Model 3	Model 1	Model 2	Model 3
	B (95% C1)		B (95% C1)	B (95% C1)	B (95% C1)	B (95% C1)	B (95% C1)	B (95% C1)	B (95% C1)	B (95% C1)
No influence of religion (Ref: Influence)	2.00*** (0.92–3.07)		1.78** (0.70–2.86)	1.57** (0.49–2.66)	3.15* (0.66–5.64)	2.13 (−0.41–4.68)	0.71 (−1.56–2.99)	1.59 (−1.48–4.61)	0.94 (−2.12–4.00)	0.30 (−2.80–3.40)
Age 40 years or less (Ref: 41 years or more)	0.50 (−0.62–1.61)		−0.83 (−1.25–1.08)	0.06 (−1.12–1.23)	0.86 (−1.69–3.41)	0.15 (−2.52–2.82)	0.30 (−2.11–2.71)	0.31 (2.77–3.39)	−0.25 –3.48–2.98	−0.12 (−3.39–3.15)
Gender, Male (Ref: Female)	−0.51 (−1.64–0.62)		0.15 (−0.97–1.28)	0.25 (−0.88–1.37)	−1.04 (−3.61–1.53)	−0.31 (−2.95–2.33)	0.61 (−1.75–2.98)	0.25 (−3.09–3.14)	0.54 (−2.63–3.70)	1.24 (−1.95–4.42)
Higher Education (Ref: Bachelor’s degree)			0.35 –0.41–1.11	0.33 (−0.42–1.08)		1.78 (−0.02–3.58)	1.75* (0.17–3.34)		−0.14 (−2.26–1.99)	−0.42 (−2.53–1.69)
More years working with SRHR (Ref: Less than 7 years)			0.07 (−0.03–0.16)	0.04 (−0.06–0.13)		0.30** (0.08–0.52)	0.20* (0.01–0.40)		0.40** (0.14–0.66)	0.29* (0.03–0.55)
Public sector employment (Ref: Private sector employment)			0.19 (−0.10–0.47)	0.15 (−0.14–0.43)		0.83* (0.16–1.50)	0.49 (−0.11–1.08)		0.83* (0.02–1.63)	0.64 (−0.17–1.45)
High SRHR self-rated knowledge (Ref: Low self-rated knowledge)				0.11* (0.01–0.22)			0.57*** (0.36–0.78)			0.41** (0.12–0.70)
(b): Association, by means of linear regression, between influence of culture on SRHR decisions and normative SRHR attitudes, active SRHR knowledge seeking and normative SRHR practice scores adjusted for participants’ characteristics.										
		Normative SRHR attitudes score			Active SRHR knowledge-seeking score			Normative SRHR practice score		
		Model 1	Model 2	Model 3	Model 1	Model 2	Model 3	Model 1	Model 2	Model 3
		B (95% C1)	B (95% C1)	B (95% C1)	B (95% C1)	B (95% C1)	B (95% C1)	B (95% C1)	B (95% C1)	B (95% C1)
No Influence of culture (Ref: Influence)		0.96 (−0.19–2.09)	0.58 (−0.57–1.73)	0.41 (−0.73–1.54)	0.17 (−0.02–0.36)	0.08 (−0.12–0.27)	0.05 (−0.13–0.22)	2.76 (−0.24–5.76)	0.98 (−2.08–4.05)	0.45 (−2.63–3.52)
Age, 40 years or less (Ref: 41 Years or more)		0.41 (−0.76–1.59)	−0.15 (−1.37–1.07)	0.06 (−1.16–1.28)	0.06 (−0.14–0.25)	−0.01 (−0.22–0.19)	−0.01 (−0.20–0.18)	0.29 (−2.76–3.35)	−0.23 (−3.36–3.00)	−0.12 (−3.38–3.15)
Gender, Male (Ref: Female)		−0.26 (−1.43–0.92)	0.41 (−0.76–1.59)	0.47 (−0.70–1.63)	−0.06 (0.26–0.13)	0.01 (−0.19–0.21)	0.07 (−0.12–0.25)	0.08 (−2.99–3.15)	0.59 -(2.55–3.74)	1.25 (−1.91–4.41)
Higher education (Ref: Bachelor’s degree)			0.53 (−0.26–1.31)	0.47 (−0.31–1.25)		0.15* (0.02–0.29)	0.15* (0.02–0.27)		−0.07 (−2.17–2.202)	−0.40 (2.50–1.69)
More years working with SRHR (Ref: Less than 7 years)			0.06 (−0.03–0.16)	0.03 (−0.07–0.13)		0.02* (0.01–0.04)	0.02 (−0.00–0.03		0.39** (0.13–0.65)	0.29* (0.02–0.55)
Public sector employment (Ref: Private sector employment)			0.24 (−0.06–0.55)	0.19 (−0.12–0.49)		0.06* (0.01–0.11)	0.04 (−0.01–0.09)		0.83* (0.03–1.63)	0.63 (−0.19–1.45)
High SRHR self-rated knowledge (Ref: Low self-rated knowledge)				0.14* (0.03–0.24)			0.04*** (0.02–0.06)			0.41** (0.13–0.700)

P value = *<0.05, **<0.01, ***<0.001

B: Unstandardized coefficient

Model 1: Adjusted for age and gender

Model 2: Adjusted for age, gender, education, years working with SRHR and employment sector

Model 3: Adjusted for age, gender, education, years working with SRHR, employment sector and SRHR self-rated knowledge

Table 4 (b) shows the association between influence of culture on SRHR decisions and the three outcome variables when adjusted for age, gender, education, years working with SRHR, employment sector and self-rated knowledge at multivariate analysis. Only high self-rated knowledge positively predicted normative SRHR attitude scores in the fully adjusted model. Although the absence of cultural influence was significantly associated with active SRHR knowledge-seeking scores in the unadjusted analysis (Table 3), no such association was found in the fully adjusted model. High level of education and high SRHR self-rated knowledge were significantly related to active SRHR knowledge-seeking scores. However, more years working with SRHR and high SRHR self-rated knowledge were statistically significant predictors of participants’ normative SRHR practice.

In the multivariate analysis shown in Table 5, the normative SRHR attitude variable was added to the list of characteristics in order to test the KAP-hypothesis. Normative SRHR attitudes were found to positively predict participants’ normative SRHR practice scores.

**Table 5.** Association, by means of linear regression, between normative SRHR attitudes scores, active SRHR knowledge-seeking scores and normative SRHR practice scores adjusted for age, gender, education and self-rated SRHR knowledge

	Normative SRHR practice scores				
		B (95% CI)	B (95% CI	B (95% CI)	B (95% CI)
	B (95% CI)	Model 1	Model 2	Model 3	Model 4
Normative SRHR attitudes (Ref: Non-normative attitudes)	1.12*** (0.65–1.59)	1.23*** (0.65–1.61)	0.65** (0.21–1.08)	0.65*** (0.23–1.08)	0.67** (0.24–1.11)
Age, 40 years or less (Ref: 41 years or more)		−0.24 (−0.315–2.67)	−0.47 (−2.94–1.99)	−0.14 (−2.59–2.33)	−0.48 (−3.00–2.03)
Gender, Male (Ref: Female)		0.66 (−2.25–3.58)	1.18 (−1.28–3.65)	0.77 (−1.70–3.23)	0.97 (−1.55–3.48)
Active SRHR knowledge seeking (Ref: Less likely to seek knowledge)			0.64*** (0.45–0.83)	0.67*** (0.47–0.86)	0.70*** (0.48–0.91)
Higher education (Ref: Bachelor’s degree)				−1.66 (−3.35–0.03)	−1.58 (−3.29–0.13)
High SRHR self-rated knowledge (Ref: Low self-rated knowledge)					−0.08 (−0.35–0.19)

Unstandardized coefficient

P value = *<0.05, **<0.01, ***<0.001

Model1: Adjusted for age and gender

Model 2: Adjusted for age, gender and active SRHR knowledge seeking

Model 3: Adjusted for age, gender, active SRHR knowledge seeking, education

Model 4: Adjusted for age, gender, active SRHR knowledge seeking, education and SRHR self-rated knowledge

No synergistic interaction was found between influence of religion (nor culture) and years working with SRHR, or between the absence of religious (or cultural) influence and employment level regarding the two outcomes: active SRHR knowledge-seeking and normative SRHR practice.

## Discussion

Health care practitioners play a crucial role towards achieving universal coverage of SRHR services. This study examined the role of individual characteristics and working environment in predicting normative SRHR attitudes and practices of healthcare practitioners. Bivariate linear regression showed statistically significant positive associations between the absence of religious influence and both normative SRHR attitudes and active SRHR knowledge-seeking practices. In a similar analysis, we also noted that number of working years with SRHR services and high SRHR self-rated knowledge positively predicted active SRHR knowledge-seeking and SRHR normative practices. Contrary to our hypothesis, reporting that religion or culture in general is important in the individual’s life did not show a statistically significant relationship to the measured SRHR attitudes and practices.

In the multivariate analysis, the statistical significance between the absence of religious influence and active knowledge-seeking was attenuated, when number of working years and SRHR self-rated knowledge were introduced as covariates in the model. Likewise, the statistically significant association between the absence of cultural influence and active SRHR knowledge-seeking at bivariate analysis disappeared in the multivariate model. Instead more years working with SRHR and higher self-rated knowledge positively predicted normative SRHR practices in the multivariate analysis. The influence of religion retained a statistically significant negative association with normative SRHR attitudes in the multivariate analysis. Regarding all the outcome variables in the full models, SRHR self-rated knowledge emerged as the strongest predictive factor, followed by number of years working with SRHR service provision. This highlights the potential of educational interventions aimed at scaling up SRHR accessibility in low resource settings.

The association between knowledge and practices among SRHR healthcare workers in low-income countries has been examined in a number of studies. For example, in a systematic review of studies conducted in Sub-Saharan Africa, poor knowledge and poor skills concerning specific SRHR components were significantly linked to inadequate provision of services [10]. However, dissociation between knowledge and practices has also been reported in other areas of health practice research e.g. high knowledge of HIV causes, transmission and prevention among populations at risk often did not correlate with adoption of prevention practices [25,26].A significant gap between awareness and uptake of contraception was reported among women of reproductive age in a scoping review on determinants of unmet need for family planning in low and middle-income countries [27], and high functional literacy and knowledge about cancer had no significant association with actual screening among patients [28].

Our study reports a very strong association between high self-rated knowledge and normative practices. However, it is plausible that knowledge, although necessary, may not be a sufficient determinant of desirable practice. In fact, when SRHR attitudes, knowledge- seeking and self-rated knowledge were examined as possible predictors of normative SRHR practice, active SRHR knowledge-seeking scores (and normative attitude scores) emerged as an independent predictor of normative SRHR practice, and the significance of self-rated knowledge diminished. This is in line with Fisher and Fisher’s supposition that the association between knowledge and practice is mediated through behavioural skills [29]. In addition, participants’ self-drive to acquire knowledge, a form of self-regulated learning [30], could be a potent trait with the potential to positively impact practitioners’ SRHR attitudes and practices. The implication is that instructors in the international training programme (and/or other SRHR educational interventions) need to receive pedagogical training in self-regulated learning theories and models to appreciate how they can maximize participants’ learning and adoption of normative attitudes and behaviours. More research is required, to examine this conceptual framework, particularly exploring the development and evaluation of self-regulated learning among healthcare practitioners in low-income countries.

Normative SRHR attitude scores strongly predicted normative SRHR practice scores in this study. This is in line with what has been reported in studies on management of drug and substance abuse in healthcare services delivery [31–34]. Studies among health practitioners working with SRHR have also reported similar findings regarding the correlation between practitioners’ attitudes with intention to provide these services [10,14,35,36]. The resultant effects, such as poor clients’ experiences, low satisfaction and reduced uptake of the services leading to poor health indicators, have also been reported [35,37]. It is important to note that, in our study, ‘no influence of religion’ on SRHR decisions and high self-rated knowledge (but not educational level) were statistically significant predictors of normative SRHR attitudes. This implies that health practitioners’ religious beliefs cannot be underestimated regardless of their perceived level of SRHR knowledge or education. Religious values are deeply held personal values for many healthcare practitioners in low-income settings to the effect that some healthcare practitioners will not participate in SRHR practices considered to be against their beliefs [6,10]. It is essential that healthcare practitioners are constantly engaged through value clarification to recognize how personal values may impact on the quality of SRHR services.

### Strengths and limitations

The main strength of this study is that it brings together a unique sample of healthcare practitioners with diverse backgrounds from medical facilities and civil society organizations, from private and public sectors, and from ten low-income countries. They included male and female persons, midwives, nurses and doctors, middle level managers, and policy makers. The study had a participation rate of 100% of the invited sample. In addition, the study is based on two validated and well-known theoretical frameworks (the Transtheoretical Model and the Knowledge-Attitude-Practices model). Although the particular items in this questionnaire (derived from these theoretical frameworks) have not been tested before, subscale analysis confirmed their reliability. However, validation of this research instrument, and the scales developed for the composite outcomes, is required.

One of the limitations of the study is the small sample size. It is likely that statistical significance was not detected for some covariates due to the small sample size. However, by choosing linear regression as the main analytical tool, we tried to maximize the value of the collected information. Furthermore, the cross-sectional nature of our study weakens its utility to make causal inference. Therefore, more studies with longitudinal or experimental study design can shed more light on our observations. It may be difficult to fully generalize our study findings to all health professionals, because this was a highly selected sample that applied for and were accepted into a capacity-building program in Sweden. However, we believe that the findings indicate general patterns among healthcare practitioners working with SRHR in similar settings.

The study was conducted at Lund University, Sweden, the host institution for the international training program. Although participants were informed that their participation in the study would not in any way affect their participation in the training program, response bias, especially social desirability bias and courtesy bias could not be completely eliminated. The most likely effect of response bias could be that attitudes and practices would be reported in a more normative manner which, in such case, would result in an underestimation of the results in this study. This was mitigated by use of a self-administered questionnaire, a method that has been reported to be less prone to response bias compared to interviewer administered instruments.

Finally, constructs like culture and religion are complex and might require more elaborate instruments. To the best of our knowledge such well-validated measures for assessing the true impact on the behaviors of individuals working in the health care sector are presently lacking regarding quantitative studies. Therefore, the lack of effect that we found in some of the analyses made in this study should be taken with adequate caution. Qualitative studies could be an alternative approach to improve this type of instruments.

## Conclusion

The findings suggest that SRHR service providers’ cultural and religious beliefs, which are often considered as obstacles for implementing full coverage of SRHR services, seem to be modified by access to information and accumulated experience over time. This highlights the importance of including elements of reflection on the role of personal values and introduction of new SRHR knowledge in the routine processes of SRHR practitioners. Interventions that introduce new knowledge, tailored to work experience, may play a key role towards universal access to high-quality SRHR services.

## Paper context

Healthcare practitioners’ attitudes and behaviours have long been perceived as a major hindrance to quality sexual and reproductive health and rights (SRHR) services in low-income countries. Our findings suggest that SRHR attitudes and behaviours are modified by active SRHR knowledge-seeking practices and accumulated working experience with SRHR. Hence interventions that introduce new knowledge, tailored to work experience, may play a key role towards universal access to high-quality SRHR services.
